# Laparoscopic sacrohysteropexy versus vaginal sacrospinous hysteropexy as treatment for uterine descent: comparison of long-term outcomes

**DOI:** 10.1007/s00192-022-05185-7

**Published:** 2022-04-28

**Authors:** Anique M.J. van Oudheusden, Anne-Lotte W.M. Coolen, Hilde Hoskam, Joggem Veen, Marlies Y. Bongers

**Affiliations:** 1 Department of Gynaecology and Obstetrics, Jeroen Bosch Hospital, Henri Dunantstraat 1, 5223 GZ s-Hertogenbosch, The Netherlands; 2 Department of Gynaecology and Obstetrics, Grow School for Oncology and Developmental Biology, Maastricht University Medical Centre+, P Debyelaan 25, 6229 HX Maastricht, The Netherlands; 3 Department of Gynaecology, Bergman Clinics, Marathon 1, 1213 PA Hilversum, The Netherlands; 4 Department of General Medicine, Maastricht University Medical Centre+, P Debyelaan 25, 6229 HX Maastricht, The Netherlands; 5 Department of Gynaecology and Obstetrics, Máxima Medical Centre, De Run 4600, 5500 MB Veldhoven, The Netherlands

**Keywords:** Uterine prolapse, Apical prolapse, Uterine descent, Sacrospinous hysteropexy, Sacrospinous ligament fixation, Laparoscopic sacrohysteropexy

## Abstract

**Introduction and hypothesis:**

Pelvic organ prolapse (POP) is a frequent occurring health issue, especially concerning elderly women. The objective of this study is to examine the long-term outcomes of laparoscopic sacrohysteropexy (LSH) and vaginal sacrospinous hysteropexy (SSHP) for treatment of uterine prolapse.

**Methods:**

A retrospective study of patients who underwent a LSH or SSHP. Validated questionnaires and an outpatient examination visit were used to investigate the effects of both surgical treatments. The primary outcome was the composite outcome of success for the apical compartment, defined as no recurrence of uterine prolapse (POP-Q measurement C ≤ 0), no subjective recurrence of POP, and/or not requiring therapy for recurrent prolapse. Secondary outcomes were peri- and postoperative data, anatomical failure, prolapse beyond hymen, subjective outcomes, and disease-specific quality of life.

**Results:**

We included 105 patients, 53 in the LSH group and 52 in the SSHP group. The overall response rate of the questionnaires was 83% (*n* = 87) after a mean follow-up time of 4.5 years (54.2 months; 95% CI 44.8–64.2 months) in the LSH group and 2.5 years (30.1 months; 95% CI 29.3–31.5 months) in the SSHP group. There were no clinically relevant differences between the study groups in composite outcome of success (*p* = 0.073), anatomical failure of the apical compartment (*p* = 0.711), vaginal bulge symptoms for which patients consulted professionals (*p* = 0.126), and patient satisfaction (*p* = 0.741). The operative time was longer in the LSH group (117 min; interquartile range (IQR) 110–123) compared to the SSHP group (67 minutes; IQR 60–73) (*p* < 0.001). The duration of hospital stay was also longer in the LSH group (4 days) than in the SSHP group (3 days) (*p* = 0.006).

**Conclusions:**

LSH and SSHP seem to be equally effective after long-term follow-up in treating uterine prolapse in terms of objective and subjective recurrence.

## Introduction

Many women suffer from pelvic organ prolapse (POP). The prevalence of POP has been reported as 40–60% in parous women [1–4]. Due to the higher life expectancy in women, the incidence of POP is expected to increase. The lifetime risk of women undergoing a single surgery for POP or urinary incontinence is 19–20% at the age of 85 [5, 6]. Vaginal hysterectomy (VH) is the most used surgical treatment worldwide for patients with symptomatic uterovaginal prolapse [7], although a hysterectomy may cause nerve damage and disrupt important supportive structures of the pelvic floor [8]. In addition, a hysterectomy alone often fails to give the right support. Recurrence of POP in women who underwent a hysterectomy for pelvic organ prolapse has been reported in 11.6% [9]. A more recent study examined the long-term prevalence of POP after hysterectomy, with a median follow-up of 16 years. The prevalence of vaginal vault prolapse was 23% in women after vaginal hysterectomy for POP, defined as POP requiring apical surgery during the follow-up period or ≥ stage 2 during POP-Q examination [10].

There is an increasing amount of evidence in favor of surgical options with uterus preservation compared to vaginal hysterectomy in the treatment of uterine descent [4, 11]. Various surgical techniques for the treatment of uterine prolapse with uterus preservation have been described, including vaginal, abdominal, and laparoscopic procedures. One of these procedures is the vaginal sacrospinous hysteropexy (SSHP). During this procedure the cervix is lifted towards one of the sacrospinous ligaments and attached with sutures, resulting in suspension of the uterus. Several studies show that SSHP is a safe procedure for the treatment of uterovaginal prolapse and severe complications are rarely seen during and after this surgery [4, 11–15]. Also, it has been shown that uterus preservation by SSHP is non-inferior to VH with suspension of the uterosacral ligaments, after a follow-up period of 12 months [4]. In a randomized controlled trial of 208 participants and a follow-up time of 5 years, significantly fewer anatomical recurrences of the apical compartment with bothersome bulge symptoms or repeat surgery were found after SSHP compared to VH with uterosacral ligament suspension. After hysteropexy a higher proportion of women had a composite outcome of success [11].

The laparoscopic sacrohysteropexy (LSH) is another surgical option for uterovaginal prolapse with uterine preservation. During a laparoscopic procedure a mesh is attached to the cervix and the other side of the mesh is fixated to the promontory by sutures or tackers to elevate the uterus. In a randomized controlled trial of 126 patients, LSH was equally effective compared to SSHP as surgical treatment of the apical compartment after 12 months of follow-up. Following LSH, bothersome overactive bladder and fecal incontinence were more frequent, but dyspareunia was reported less frequently. However, the follow-up time in this publication of the trial was only 12 months [15].

Both uterovaginal suspension techniques seem to be an effective procedure with low risk of complications for patients with uterovaginal prolapse. However, evidence comparing LSH to SSHP for uterine prolapse with long-term follow-up is lacking. We wondered what the long-term effects of LSH and SSHP would be and therefore performed a retrospective trial with a long-term follow-up and evaluated whether one of the two surgeries is preferable to treat apical prolapse.

## Materials and methods

### Study design

We performed a retrospective cohort study in the Máxima Medical Centre (MMC), a teaching hospital in The Netherlands. The ethical research committee of the MMC waived the need for approval (file number 2014-12). After assessment of the study protocol, the committee judged that the rules laid down in the medical Research Involving Human Subjects Act did not apply to this research proposal. This study was developed and described in accordance with the strengthening the reporting of observational studies in epidemiology (STROBE) guidelines [16]. The results are reported by means of the IUGA/ICS recommendations for reporting outcomes of surgical procedures for pelvic organ prolapse [17].

The study population consisted of patients who underwent a LSH between 2003 and 2013 or a SSHP between 2009 and 2011 for primary treatment of uterine prolapse. The SSHP was introduced in our hospital in 2009. Both techniques were performed by experienced gynecologists, who had completed their learning curves. If indicated, the LSH or SSHP was combined with concomitant surgery such as an anterior or posterior colporrhaphy (both performed vaginally) or a mid-urethral sling (MUS). Additional surgery was included in the duration of the operative time. The choice of treatment was left to the discretion of the gynecologist and based on the preference of the patient and gynecologist. Patients with a history of hysterectomy were excluded.

### Outcome measures

The primary outcome was the composite outcome of success for the apical compartment, defined as no recurrence of uterine prolapse (POP-Q measurement C ≤ 0) [18, 19], no bothersome bulge symptoms, and/or not requiring retreatment for recurrent prolapse (either surgery or conservative treatment) [20]. A positive answer on any of the following questions of the Urinary Distress Inventory (UDI) questionnaire is scored as a subjective recurrence: ‘Do you experience a sensation of bulging or protrusion from the vagina?’ and ‘Do you have a bulge or something protruding that you can see in the vagina?’ in combination with a response ‘slightly bothersome’ to ‘greatly bothersome’ to the question ‘how much does this bother you?’

Secondary outcomes were anatomical failure (POP-Q ≥ stage 2 in any compartment), prolapse beyond hymen (POP-Q measurements > 0), reinterventions, subjective outcomes, and disease-specific quality of life. Furthermore, patient characteristics, preoperative morbidity, postoperative complications, and follow-up data were evaluated.

### Surgical interventions

The laparoscopic sacrohysteropexy (LSH) was performed under general anesthesia. A uterine manipulator was used. After insufflation, four laparoscopic ports were placed: one 10-mm umbilical, two 5-mm lateral ports, and one 12-mm disposable trocar suprapubic. The ureter was identified on the right side. The peritoneum was incised from the sacral promontory to the level of the cervico-uterine junction. The vesico-uterine peritoneum was incised, and the bladder was dissected from the cervix. A bifurcated polypropylene type-1 monofilament microporous non-absorbable mesh was fixated to the posterior side of the cervix with four sutures. An inverted Y-shaped mesh was attached with four sutures to the anterior side of the cervix. Both ends of the Y-shaped mesh were perforated through the broad ligament and sutured to the posterior mesh, dorsally of the uterus. The end of the posterior mesh was attached to the sacral promontory using staples and was peritonealized.

The sacrospinous hysteropexy (SSHP) was conducted under general or spinal anesthesia. After hydrodissection, the posterior vaginal wall was opened and the right sacrospinous ligament was exposed by blunt dissection, via the pararectal space. Breisky retractors were inserted for clear vision of the ligament. Two non-absorbable sutures were passed through the sacrospinous ligament, 2 cm medial to the ischial spine. Then, the sutures were placed through the posterior side of the cervix, resulting in suspension of the uterus. The vaginal wall was closed with absorbable sutures. Concomitant anterior or posterior vaginal wall repair or anti-incontinence surgery was performed if indicated with either the LSH or the SSHP.

### Peri- and postoperative care

All patients were given a transurethral catheter and antibiotic prophylaxis (cefazolin and metronidazole) during surgery. The catheter was removed the next day or after the second day in case of an anterior colporrhaphy. After a SSHP, an intravaginal gauze packing was placed until the next day to reassure hemostasis. Thrombosis prophylaxis (subcutaneous injection of low molecular weight heparin) was prescribed during admission.

All patients were seen for follow-up 6 weeks after surgery as part of regular postoperative care. Evaluation of the POP symptoms and anatomical results, using the Pelvic Organ Prolapse Quantification (POP-Q), were registered. Information from possible additional follow-up visits was acquired from patients’ files.

### Data collection

In 2014, 2 to 11 years after POP surgery, all women who had undergone a SSHP or a LSH were contacted by mail and asked to fill in various Dutch validated questionnaires. Disease-specific quality of life was tested by the Patient Global Impression of Improvement (PGI-I) [21], Urinary Distress Inventory (UDI) [22], Defecatory Distress Inventory (DDI) [23], and Incontinence Impact Questionnaire (IIQ) [22]. The UDI and DDI, containing of 19 and 11 items, respectively, indicate whether complaints of micturition, prolapse, or defecation are present and to what extent they are bothersome. These questions are designed using a four-point Likert scale ranging from ‘not at all’ to ‘greatly’. The IIQ consists of 13 questions and shows the disease-specific quality of life for urine incontinence, also using a four-point Likert scale. The score of each domain ranges from 0 to 100; high score indicates increasingly bothersome symptoms (UDI and DDI) and a poorer quality of life (IIQ).

Furthermore, we evaluated sexual functioning, using the Prolapse/Incontinence Sexual Questionnaire (PISQ), containing 12 questions. The PISQ covers three domains: behavioral-emotive, physical, and partner-related. These items are scored on a five-point Likert scale ranging from 0 (always) to 4 (never). Items 1–4 are reversely scored, and a total of 48 is the maximum score; higher scores indicate better sexual function [24, 25].

If patients did not return the questionnaires, we contacted them by telephone and sent the questionnaires again. The follow-up time for patients was recorded from surgery until completion of the questionnaire. For the non-responders, the follow-up time was calculated from surgery until the time of the last data collection from patients’ files.

The follow-up consult consisted of an evaluation of prolapse symptoms and possible long-term complications combined with a vaginal examination to evaluate anatomical results using the POP-Q. Long-term complications included suture and mesh exposures as well as chronic pain symptoms. We asked the patient whether they had consulted a physician because of prolapse-related complaints or had a retreatment elsewhere. These follow-up visits were performed by a researcher, who was trained and authorized for POP-Q examination. The gynecologists who had performed the POP surgeries were not involved in the evaluation in order to maintain objectivity.

### Statistical analysis

The LSH group and SSHP group were compared using the Student’s *t*-test for normally distributed data and the Mann-Whitney test for skewed data. For categorical data, the chi-square and Fisher tests were used. Follow-up time, age, and preoperative stage of uterine prolapse were evaluated as confounders in a logistic and linear regression analysis. Changes for > 10% in Exp(B) or β were viewed as confounding and further investigated in multivariable regression analysis. The statistical analysis was completed in Statistical Package for the Social Sciences (SPSS) 25.0 database for Windows.

## Results

**Fig. 1:**
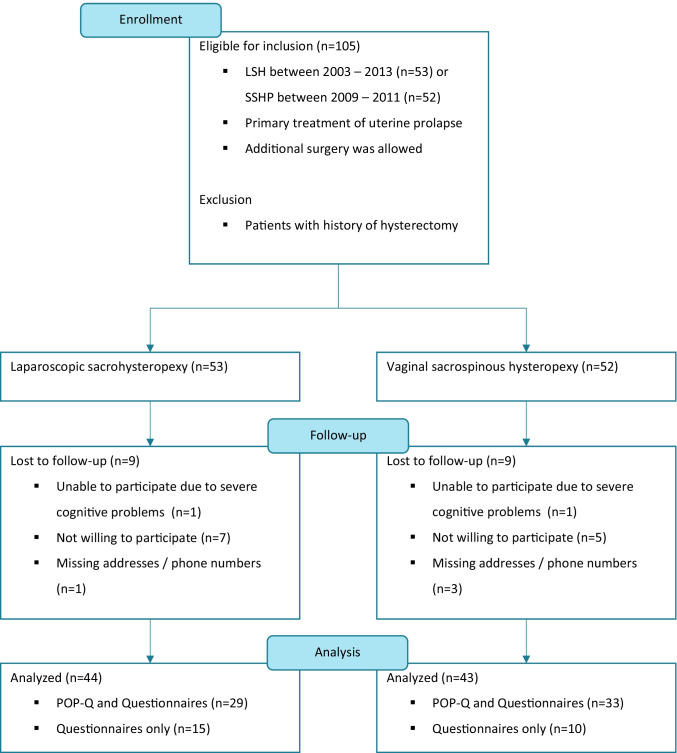
Flowchart of the study population

One hundred five patients were eligible for inclusion: 53 in the LSH group and 52 in the SSHP group, as shown in Fig. 1. The questionnaires were completed by 44 (83.0%) patients in the LSH group and 43 (82.7%) patients in the SSHP group. Twenty-nine (54.7%) patients from the LSH group and 33 (63.5%) from the SSHP group came to our outpatient clinic for a POP-Q examination. Eighteen (17.1%) patients were lost to follow-up for various reasons; 2 patients had severe cognitive problems, 4 patients could not be contacted because of missing addresses or telephone numbers, and 12 patients were not willing to participate in a follow-up study. The mean follow-up time of this study is 4.5 years (54.2 months; 95% CI 44.8–64.2 months) in the LSH group and 2.5 years (30.1 months; 95% CI 29.3–31.5 months) in the SSHP group, which is significantly different, *p* < 0.001.

Table 1 shows the baseline characteristics of the study population. It shows significant differences in age at time of surgery as well as the age at time of follow-up. The LSH group was younger, with a mean age of 52.2 (95% CI 48.8–22.6) years at baseline and 56.7 (95% CI 53.2–60.3) years at follow-up compared to the SSHP group, whose the mean age was 60.7 years (95% CI 57.3–64.1) at baseline and 63.7 years (95% CI 60.0–67.3) at follow-up, *p* = 0.001 and *p* = 0.008, respectively. In the LSH group there were more participants with a POP-Q stage ≥ 3 of the apical and anterior compartments compared to the SSHP group, *p* = 0.006 and *p* = 0.003, respectively. There was no difference in stage of posterior vaginal wall prolapse at baseline, *p* = 0.125.

**Table 1:** Baseline characteristics

Characteristics	Laparoscopic sacrohysteropexy (*n* = 53)	Vaginal sacrospinous hysteropexy (*n* = 52)	*p*-value
Age during surgery (years)			***0.001***^***a***^
Mean (95% CI)	52.2 (48.8–55.6)	60.7 (57.3–64.1)	
Age at follow-up (years)			***0.008*** ^***a***^
Mean (95% CI)	56.7 (53.2–60.3)	63.7 (60.0–67.3)	
Parity No./total no. of patients (%)			*0.792*^*b*^
0	2/42 (4.8)	1/43 (2.3)	
1	3/42 (7.1)	3/43 (7.0)	
2	26/42 (61.9)	27/43 (62.8)	
3	10/42 (23.8)	9/43 (20.9)	
≥ 4	1/42 (2.4)	3/43 (7.0)	
Body mass index (kg/m^2^**)**			*0.835*^*a*^
Mean (95% CI)	24.9 (23.9–26.0)	25.1 (24.1–26.2)	
History of gynecological surgery no./total no. of patients (%)			*0.234*^*b*^
None	39/53 (73.6)	46/52 (88.4)	
ACR/PCR	10/53 (18.9)	6/52 (11.5)	
Vaginal sacrospinous hysteropexy	2/53 (3.8)	-	
Laparoscopic sacrohysteropexy	1/53 (1.9)	-	
Manchester Fothergill	1/53 (1.9)	-	
POP-Q stage apical compartment (point C) No./total no. of patients (%)			***0.006***^***b***^
1	2/52 (3.8)	5/51 (9.8)	
2	23/52 (44.2)	34/51 (66.7)	
3	20/52 (38.5)	12/51 (23.5)	
4	7/52 (13.5)	0/51 (0.0)	
POP-Q stage anterior compartment (point Ba) No./total no. of patients (%)			***0.003***^***b***^
0	4/44 (9.1)	5/48 (10.4)	
1	7/44 (15.9)	0/48 (0.0)	
2	7/44 (15.9)	22/48 (45.8)	
3	25/44 (56.8)	21/48 (43.8)	
4	1/44 (2.3)	0/48 (0.0)	
POP-Q stage posterior compartment (point Bp) No./total no. of patients (%)			*0.125*^*b*^
0	7/38 (18.4)	19/45 (42.2)	
1	15/38 (39.5)	10/45 (22.2)	
2	11/38 (29.0)	12/45 (26.7)	
3	5/38 (13.2)	5/45 (11.1)	
Duration of follow-up (months)			***< 0.001***^***a***^
Mean (95% CI)	54.2 (44.8–64.2)	30.1 (29.3–31.5)	

ACR = anterior colporrhaphy; PCR = posterior colporrhaphy

^*a*^Student’s *t*-test

^*b*^Pearson’s chi-square

Table 2 displays the perioperative data and complications. The LSH group had a significantly longer mean surgery time of 117 (IQR 110-123) min compared to the SSHP group, which had a mean operative time of 67 (IQR 60-73) min, *p* < 0.001. Mean estimated amount of blood loss in the LSH group was less, 60 (95% CI 44-74) ml compared to the blood loss in the SSHP group of 168 (95% CI: 131-205) ml, *p* < 0.001. Duration of hospital stay in the LSH group was significantly longer than in the SSHP group, 4 versus 3 days, *p* = 0.006.

**Table 2:** Perioperative data and complications

	Laparoscopic sacrohysteropexy (*n* = 53)	Vaginal sacrospinous hysteropexy (*n* = 52)	*p*-value
Operative time (minutes) Median (IQR)	117 (110–123)	67 (60–73)	***< 0.001***^***c***^
Estimated blood loss (ml) Mean (95% CI)	60 (44–74)	168 (131–205)	***< 0.001***^***a***^
Hospital stay (days) Mean (95% CI)	4 (3.5–4.2)	3 (3.0–3.5)	***0.006***^***a***^
Perioperative complications No./total no. of patients (%)			*0.396*^*b*^
One or more complications	2/53 (3.8)	1/52 (1.9)	
Bleeding	1/53 (1.9)	-	
Alternative mesh fixation	1/53 (1.9)	-	
Anesthesia-induced complication	-	1/52 (1.9)	
Concomitant surgery No./total no. of patients (%)	17/53 (32.1)	46/52 (88.5)	*< 0.001*^*b*^
None	36/53 (67.9)	6/52 (11.5)	
ACR	-	33/52 (63.5)	
PCR	1/53 (1.9)	3/52 (5.8)	
ACR + PCR	-	4/52 (7.5)	
Perineorrhaphy	3/53 (5.7)	-	
ACR + perineorrhaphy	-	3/52 (5.8)	
PCR + MUS	-	1/52 (1.9)	
ACR + PCR + MUS	-	1/52 (1.9)	
VM	-	1/52 (1.9)	
MUS	3/53 (5.7)	-	
Other than for POP/UI	9/53 (17.0)	-	
Postoperative complications < 2 weeks No./total no. of patients (%)			*0.458*^*b*^
One or more complications	7/53 (13.2)	12/52 (23.1)	
Ileus	1/53 (1.9)	-	
Abdominal wall hematoma	1/53 (1.9)	-	
Retroperitoneal hematoma	-	1/52 (1.9)	
Urinary tract infection	2/53 (3.8)	1/52 (1.9)	
Urinary retention (> 150 ml)	2/53 (3.8)	7/52 (13.5)	
Anemia	-	4/52 (7.5)	
Postoperative complications > 2 weeks No./total no. of patients (%)			*0.381*^*b*^
One or more complications	12/53 (22.6)	9/52 (17.3)	
Recurrent urinary tract infection	-	2/52 (3.8)	
Urinary retention (> 150 ml)	1/53 (1.9)	1/52 (1.9)	
De novo dyspareunia	1/53 (1.9)	3/52 (5.8)	
Bottom pain	-	2/52 (3.8)	
Constipation	2/53 (3.8)	-	
Abdominal pain	6/53 (11.3)	-	
Exposure mesh	3/53 (5.7)	-	
Exposure sutures	-	1/52 (1.9)	

ACR = anterior colporrhaphy; PCR = posterior colporrhaphy; MUS = mid-urethral sling; VM = vaginal mesh (Prolift anterior); POP = pelvic organ prolapse; UI = urine incontinence

^*a*^Student’s *t*-test

^*b*^Pearson’s chi-square

^*c*^Mann-Whitney test

There was significantly less perioperative concomitant surgery in the LSH group (32.1% additional surgery) compared to the SSHP group (88.5% additional surgery), *p* < 0.001. In the LSH group there were four additional procedures because of POP complaints and three procedures because of stress urine incontinence. In nine cases (17%) the concomitant surgery was not related to POP complaints; four patients (7.5%) had a sterilization with Filshie-clips; three patients (5.7%) had a bilateral salpingo-oophorectomy; one patient (1.9%) had a correction of an abdominal herniation; 1 patient (1.9%) had a hysteroscopic polypectomy. In the SSHP group 41 patients (78.8%) had an anterior colporrhaphy as concomitant surgery; in 8 cases (15.4%) this was combined with a posterior colporrhaphy, perineorrhaphy and/or mid-urethral sling. Three patients (5.8%) had a posterior colporrhaphy; one patient (1.9%) had a posterior colporrhaphy combined with a mid-urethral sling; one patient (1.9%) had a vaginal mesh (Prolift anterior).

Like the perioperative complications, the postoperative complications were not significantly different. Mesh exposure happened during the follow-up period after a LSH in three cases (5.7%). In all cases excision of the exposure was necessary. In one participant (1.9%) of the SSHP group, the sutures were visible and needed to be shortened in the outpatient clinic. Dyspareunia de novo occurred in one patient (1.9%) in the LSH group versus three patients in the SSHP group (5.8%), *p* = 0.299. In six (11.3%) of the women in the LSH group, chronic abdominal pain was reported, whereas in the SSHP group none of the participants said they had this complaint. One patient already had abdominal complaints before surgery, one patient had heavy menstrual bleeding due to uterine fibroids, one patient had irritable bowel syndrome, and three patients reported de novo abdominal pain (5.7%).

**Fig. 2:**
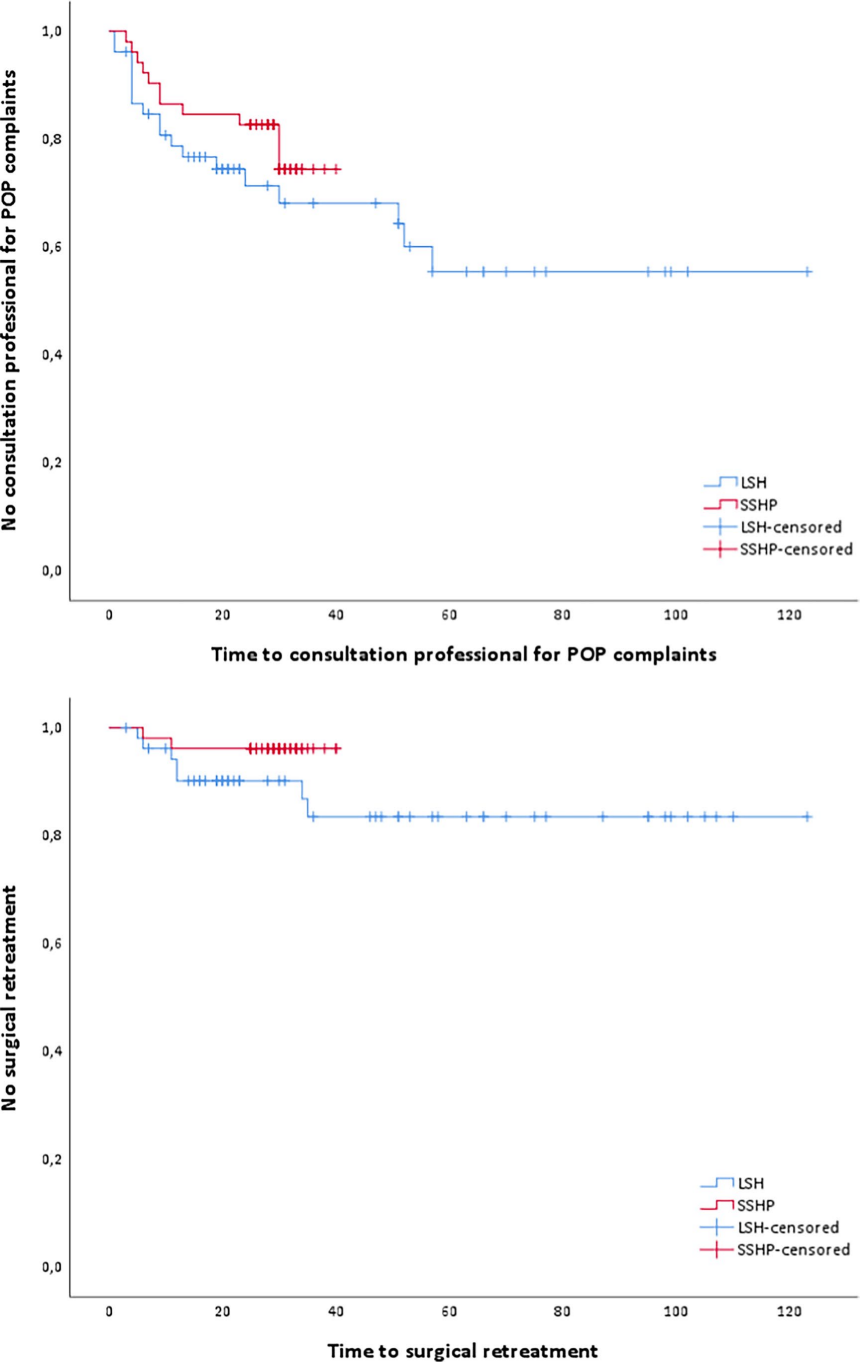
Survival analysis of consultation professional for POP complaints and surgical retreatment. Time (months) to consultation professional for POP complaints, *p* = 0.306. Time (months) to surgical retreatment, *p* = 0.164

The composite outcome of success for the apical compartment was 41.4% in the LSH group compared to 72.7% in the SSHP group (*p* = 0.073), as is shown in Table 3. There were no significant differences between the study groups concerning anatomical failure of the apical compartment in long-term follow-up (*p* = 0.711). Also, conservative and surgical re-interventions show no significant differences between the two study groups; *p* = 0.158 and *p* = 0.242, respectively (Fig. 2). Regression analysis for composite outcome of success and anatomical failure showed no confounding for duration of follow-up, age, or preoperative POP-Q stage of uterine descent.

**Table 3:** Recurrences of POP and retreatment after surgery

	Laparoscopic sacrohysteropexy (*n* = 53)	Vaginal sacrospinous hysteropexy (*n* = 52)	*p*-value
Composite outcome of success No./total no. of patients (%)	12/29 (41.4)	24/33 (72.7)	*0.073*^*b*^
Anatomical failure No./total no. of patients (%)			
During follow-up consultation at 6 weeks			
Anterior compartment (Ba ≥ -1)	3/26 (11.5)	6/45 (13.3)	*0.827*^*b*^
Apical compartment (C ≥ -1)	0/33 (0.0)	0/46 (0.0)	*-*
Posterior compartment (Bp ≥ -1)	4/17 (23.5)	1/33 (3.0)	***0.040***^***b***^
During follow-up consultation at long-term follow-up			
Anterior compartment (Ba ≥ -1)	25/28 (89.3)	25/33 (75.8)	*0.171*^*b*^
Apical compartment (C ≥ -1)	1/28 (3.6)	1/33 (3.0)	*0.711*^*b*^
Posterior compartment (Bp ≥ -1)	6/26 (23.1)	0/33 (0.0)	***0.006***^***b***^
Prolapse beyond hymen No./total no. of patients (%)			
During follow-up consultation at 6 weeks			
Anterior compartment (Ba > 0)	1/26 (3.8)	3/45 (6.7)	*0.619*^*b*^
Apical compartment (C > 0)	0/33 (0.0)	0/46 (0.0)	*-*
Posterior compartment (Bp > 0)	2/17 (11.8)	0/33 (0.0)	***0.044***^***b***^
During follow-up consultation at long-term follow-up			
Anterior compartment (Ba > 0)	16/29 (55.2)	17/33 (51.5)	*0.773*^*b*^
Apical compartment (C > 0)	1/29 (3.4)	0/33 (0.0)	*0.282*^*b*^
Posterior compartment (Bp > 0)	3/27 (11.1)	0/33 (0.0)	***0.049***^***b***^
Vaginal bulge symptoms No./total no. of patients (%)			
Symptoms for which patient consulted professional	18/52 (34.6)	11/52 (21.2)	*0.126*^*b*^
Time to consulted professional (months) Median (IQR)	22.0 (10.5–55.0)	28.0 (25.0–31.0)	*0.306*^*d*^
Recurrence POP on UDI questionnaire*	16/43 (37.2)	6/41 (14.6)	***0.019***^***b***^
Conservative retreatment No./total no. of patients (%)	15/52 (28.8)	6/52 (11.5)	*0.158*^*b*^
Physical therapy	12/52 (23.1)	5/52 (9.6)	
Pessary treatment	2/52 (3.8)	1/52 (1.9)	
Combined	1/52 (1.9)	0/52 (0.0)	
Surgical retreatment No./total no. of patients (%)	7/53 (13.2)	2/52 (3.8)	*0.242*^*b*^
SSHP + ACR + PCR	1/53 (1.9)	-	
VH + ACR	1/53 (1.9)	-	
VH	-	1/52 (1.9)	
MUS	1/53 (1.9)	-	
ACR	2/52 (3.8)	-	
VM	2/52 (3.8)	-	
Manchester Fothergill + ACR	-	1/52 (1.9)	
Time to surgical re-intervention (months) median (IQR)	12.0 (6.9–34.4)	9.2 (5.2–11.5)	*0.164*^*d*^

*Bulge symptoms on UDI questionnaire: ‘Slightly bothersome’ to ‘greatly bothersome’

SSHP = vaginal sacrospinous hysteropexy; ACR = anterior colporrhaphy; PCR = posterior colporrhaphy; VH = vaginal hysterectomy; MUS = mid-urethral sling; VM = vaginal mesh (Prolift anterior)

^*a*^Student’s *t*-test

^*b*^Pearson’s chi-square

^*d*^Log-rank test

In the LSH group, 18 patients (34.6%) consulted a physician because of prolapse-related complaints (in our hospital or elsewhere) as opposed to 11 patients (21.2%) in the SSHP group (*p* = 0.126), according to the questionnaire. Sixteen versus six patients in the LSH and SSHP groups (37.2% versus 14.6%), respectively, reported recurrence of POP according to the UDI questionnaire (*p* = 0.019). Subsequently, vaginal bulge symptoms occurred significantly more in the LSH group; recurrence of POP had an odds ratio (OR) of 3.46 when comparing LSH to SSHP (*p* = 0.022). However, after correcting for the confounder duration of follow-up the OR was 2.73 and not statistically significant (*p* = 0.080).

The POP-Q results, after a mean follow-up duration of 4.5 years (54.2 months) in the LSH group and 2.6 years (30.1 months) in the SSHP group are shown in Table 4. Point Bp on the posterior wall of the vagina was significantly more descended in the LSH group (mean –2.2; SD ± 1.1) compared to the SSHP group (mean –2.8; SD ± 0.4) (*p* = 0.031). The difference in the posterior compartment between the two groups was present at 6 weeks follow-up as well as during long-term follow-up. Point Bp was positioned more cranially in the SSHP group compared to the LSH group based on linear regression (β –0.111, *p* = 0.051). No confounders were found. The other points of the POP-Q were not statistically different. Point C was –6.2, SD ± 1.7 in the LSH group versus 6.0, SD ± 1.5 in the SSHP group, *p* = 0.501.

**Table 4:** POP-Q long-term follow-up

	Laparoscopic sacrohysteropexy (*n* = 27)	Vaginal sacrospinous hysteropexy (*n* = 33)	*p*-value ^c^
Aa	-0.4 ± 1.8 (-3–3)	-0.6 ± 1.4 (-3–3)	*0.559*
Ba	-0.2 ± 1.5 (-3–5)	0.6 ± 2.3 (-3–4)	*0.580*
C	-6.2 ± 1.7 (-8–0)	-6.0 ± 1.5 (-8–-1)	*0.501*
GH	3.7 ± 0.7 (2–5)	3.7 ± 0.7 (2–5)	*0.888*
PB	3.0 ± 0.2 (3–4)	3.0 ± 0.2 (3–4)	*0.865*
TVL	9.4 ± 0.7 (8–11)	9.5 ± 0.7 (9–12)	*0.626*
Ap	-2.3 ± 1.0 (-3 – 0)	-2.8 ± 0.4 (-3 – -2)	*0.077*
Bp	-2.2 ± 1.1 (-3–0)	-2.8 ± 0.4 (-3–-2)	***0.031***
D	-7.4 ± 1.6 (-9–-2)	-7.3 ± 1.3 (-9–-3)	*0.522*

Data are means ± standard deviation (range lowest–highest)

^c^Mann Whitney-U

The PGI-I in Table 5 did not show a difference in patient satisfaction; 75.0% (*n* = 33) of patients in the LSH group said their postoperative condition is ‘very much better’ or ‘much better’ now compared to 71.8% (*n* = 28) in the SSHP group (*p* = 0.741). The disease-specific quality of life from the UDI questionnaire showed a significantly higher score in the domain ‘genital prolapse’ in the LSH group with a mean score of 13.8 versus a mean score of 5.4 in the SSHP group (β -8.35, *p* = 0.044). After correcting for the confounder duration of follow-up time in linear regression analysis, the β was -4.11 and not significantly different between the two study groups, *p* = 0.316. Age and POP-Q stage of utero vaginal prolapse were not confounders. More patients in the LSH group were sexually active compared to the SSHP group, 83.3% and 56.1%, respectively (*p* = 0.007). For the sexually active women a PISQ score was calculated, which showed a total score of 37.4 in the LSH group and 36.8 in the SSHP group (*p* = 0.722). Also in the three subdomains of the PISQ no statistical differences were found between the two groups.

**Table 5:** Domain scores for disease-specific quality of life

	Laparoscopic sacrohysteropexy (*n* = 53)	Vaginal sacrospinous hysteropexy (*n* = 52)	*p*-value
Patient satisfaction (PGI-I) No./total no. of patients (%)			
‘Very much better’ or ‘much better’	33/44 (75.0)	28/39 (71.8)	*0.741*^*b*^
Urogenital distress inventory (UDI)			
Overactive bladder	8.3 (3.8–12.9)	13.7 (7.4–19.9)	*0.165*^*a*^
Urinary incontinence	7.5 (4.2–10.9)	9.8 (5.0–14.6)	*0.425*^*a*^
Obstructive micturition	7.7 (3.5–12.0)	11.0 (4.9–17.0)	*0.369*^*a*^
Genital prolapse	13.8 (7.2–20.3)	5.4 (0.5–10.3)	*0.044*^*a*^
Pain	17.1 (10.2–23.9)	8.8 (2.5–15.1)	*0.081*^*a*^
Defecatory distress inventory (DDI)			
Obstipation	5.6 (1.8–9.3)	4.9 (1.5–8.3)	*0.798*^*a*^
Obstructive defecation	7.5 (3.3–11.8)	4.8 (0.6–8.6)	*0.324*^*a*^
Pain	3.7 (1.3–7.6)	2.4 (0.8–5.2)	*0.610*^*a*^
Fecal incontinence	4.8 (1.1–8.4)	3.3 (0.8–7.7)	*0.610*^*a*^
Incontinence impact questionnaire (IIQ)			
Physical	8.5 (3.7–13.4)	10.4 (3.5–17.2)	*0.657*^*a*^
Mobility	7.1 (3.9–10.2)	12.0 (5.4–18.7)	*0.176*^*a*^
Social	4.0 (1.2–6.8)	6.9 (1.3–12.4)	*0.353*^*a*^
Embarrassment	5.3 (1.7–8.9)	9.3 (1.8–16.7)	*0.334*^*a*^
Emotional	6.9 (2.9–11.0)	10.2 (3.3–17.1)	*0.405*^*a*^
General quality of life	83.6 (79.8–87.5)	78.4 (73.4–83.5)	*0.097*^*a*^
Sexual function			
Sexually active No./total no. of patients (%)	35/42 (83.3)	23/41 (56.1)	***0.007***^***b***^
PISQ-12 Total score	37.4 (35.5–39.3)	36.8 (33.9–39.7)	*0.722*^*a*^
Behavioral-emotive	11.3 (10.2–12.3)	11.1 (9.6–12.4)	*0.874*^*a*^
Physical	17.4 (16.3–18.5)	16.7 (15.0–18.4)	*0.482*^*a*^
Partner-related	9.1 (8.6–12.4)	8.6 (7.8–9.3)	*0.214*^*a*^

All data are means (95% confidence interval), unless otherwise indicated

UDI and DDI; each item: 0 = no bothersome symptoms; 100 = most bothersome symptoms

IIQ; each item: 0 = best quality of life; 100 = worst quality of life

PISQ-12 Total score: 0 = worst sexual function; 48 = best sexual function

PISQ-12 Behavioral-emotive (items 1–4): 0 = worst function; 16 = best function

PISQ-12 Physical (items 5–9): 0 = worst function; 20 = best function

PISQ-12 Partner-related (items 10–12): 0 = worst function; 12 = best function

^*a*^Student’s *t*-test

^*b*^Pearson’s chi-square

## Discussion

### Main findings

We performed a retrospective study in a teaching hospital in The Netherlands and included 105 patients who underwent a LSH or a SSHP for uterine prolapse. After correcting for confounding factors, LSH and SSHP seem to be equally effective in treating uterine prolapse, composite outcome measures, and reported vaginal bulge symptoms. There were no clinically relevant differences in terms of anatomic recurrence of apical prolapse, POP symptoms for which patients consulted professionals, re-operation rates, and disease-specific quality of life. The operative time and hospital stay were significantly longer in the LSH group, whereas the estimated blood loss was more in the SSHP group.

Surgery time was longer for the LSH procedure, despite the higher rates of concomitant surgery that was performed during SSHP for the other compartments. This finding was to be expected and correlates to the literature, since LSH is a more complex laparoscopic procedure [2]. Blood loss was significantly less during LSH. However, the difference between the two groups was only around 100 ml estimated blood loss; therefore, it is not clinically relevant. This amount of blood loss is concordant with other literature [2, 26]. Hospital stay was longer in the LSH group (4 days) compared to the SSHP group (3 days). Since the procedures were performed, between 2003 and 2013, hospital protocols in The Netherlands have been changed, and admission after these surgeries is usually shorter nowadays.

The risk of vaginal mesh exposure in our study for the LSH group was 5.7% (*n* = 3) over a mean follow-up time of 4.5 years. In the literature, mesh exposure of 1–3% after LSH has been reported [27]. This lower incidence of mesh exposure is probably due to a shorter period of follow-up. Our follow-up time is much longer and exposures occur more often after a longer period of follow-up, as is seen in a study with 7-year follow-up and exposure rate of 10.5% [28].

In six (11.3%) of the women of the LSH group, chronic abdominal pain was reported, whereas in the SSHP group none of the participants stated to have this complaint. However, only half of those patients reported de novo abdominal pain (5.7%); in three patients a different cause was identified. The abdominal pain was not severe enough to perform a diagnostic laparoscopy or refer to another specialty (e.g., a surgeon). Other studies did not report on abdominal pain as a long-term outcome measure [15, 28–31].

Postoperatively, point Bp on the posterior wall of the vagina had descended significantly more in the LSH group compared to the SSHP group. The difference in the posterior compartment was already present at 6-week follow-up. However, the difference for point Bp is only 0.6 cm, and just one re-operation was done for the posterior compartment in the LSH group, which suggests that the difference in the posterior compartment might not be clinically relevant. Also, in a randomized controlled trial there seems to be a difference in anatomical failure for the posterior compartment (LSH 18.2% versus SSHP 6.9%). Although this is not statistically significant, it may show a trend and corresponds to our results [15].

### Strengths and limitations

The strength of this study is that the composite outcome of success of the apical compartment has been used as primary outcome. Barber’s publication underlines the importance of an outcome which includes objective and subjective POP correction as well as retreatment [20]. Another strength is the long follow-up time of 4.5 years (54.2 months; 95% CI 44.8–64.2 months) in the LSH group and 2.6 years (30.1 months; 95% CI 29.3–31.5 months) in the SSHP group. There are no prospective comparative studies which have comparable length of follow-up [15]. To evaluate the effectiveness of a surgical treatment for POP, a long follow-up time is desirable [20].

There are also some limitations to this study. Due to the retrospective design, the two study groups are significantly different regarding the baseline characteristics and the duration of follow-up time. However, the primary outcome regression models were used to eliminate three confounders: duration of follow-up, age, and stage of POP. In addition, patient selection occurred, since in our clinic SSHP is less likely to be performed in young sexually active women compared to older postmenopausal women given the higher rate of dyspareunia de novo after SSHP. Also, there is a time difference between the two surgical procedures of > 5 years (LSH 2003–2013 and SSHP 2009–2011), which can influence the study results. Although we believe this is not the case in our study, it is an important aspect to address. All surgeons had completed their learning curve before the start of our study. Moreover, during this period there were no significant changes or improvements in surgical equipment or procedures, expecting better outcomes.

In the LSH group more women were sexually active compared to the SSHP group, postoperatively. The difference in sexual activity might be explained by the significantly lower age in the LSH group. De novo dyspareunia occurred in both groups, but showed no statistically difference: 1.9% (*n* = 1) in the LSH group versus 5.8% (*n* = 3) in the SSHP group (*p* = 0.299). A randomized controlled trial on the topic found more dyspareunia after the SSHP [15]. The PISQ scores also showed no significant differences between the groups.

## Conclusion

In conclusion, the LSH and SSHP are equally effective based on objective and subjective recurrence rates, after correction for confounding factors. The operative time and hospital stay were significantly longer in the LSH group, whereas the estimated blood loss was more in the SSHP group. Peri- and postoperative complications are equal. The risk of vaginal mesh exposure is 3.8% after a mean follow-up time of 54.2 months. LSH as a treatment for uterine descent is promising; however, the long-term follow-up of a randomized controlled trial is needed to compare the effectiveness of these interventions for uterine prolapse.

## Abbreviations

POP: Pelvic organ prolapse
POP-Q: Pelvic organ prolapse Quantification
LSH: Laparoscopic sacrohysteropexy
SSHP: Sacrospinous hysteropexy
ACR: Anterior colporrhaphy
PCR: Posterior colporrhaphy
MUS: Mid-urethral sling
VH: Vaginal hysterectomy
UDI: Urinary Distress Inventory
DDI: Defecatory Distress Inventory
IIQ: Incontinence Impact Scale
PGI-I: Patient Global Impression of Improvement
PISQ: Prolapse/Incontinence Sexual Questionnaire
